# Modeling temperature- and Cav3 subtype-dependent alterations in T-type calcium channel mediated burst firing

**DOI:** 10.1186/s13041-021-00813-7

**Published:** 2021-07-17

**Authors:** Fernando R. Fernandez, Mircea C. Iftinca, Gerald W. Zamponi, Ray W. Turner

**Affiliations:** 1 Department of Biomedical Engineering, Boston University, Boston, MA USA; 2 Department of Cell Biology and Anatomy, Cumming School of Medicine University of Calgary, Calgary, Canada; 3 Department of Physiology and Pharmacology, Cumming School of Medicine University of Calgary, Calgary, Canada; 4 Hotchkiss Brain Institute, University of Calgary, Calgary, Canada; 5 Alberta Children’s Hospital Research Institute, University of Calgary, Calgary, Canada

**Keywords:** Cav3, T-type, Calcium channel, Rebound burst discharge, Temperature

## Abstract

T-type calcium channels are important regulators of neuronal excitability. The mammalian brain expresses three T-type channel isoforms (Cav3.1, Cav3.2 and Cav3.3) with distinct biophysical properties that are critically regulated by temperature. Here, we test the effects of how temperature affects spike output in a reduced firing neuron model expressing specific Cav3 channel isoforms. The modeling data revealed only a minimal effect on baseline spontaneous firing near rest, but a dramatic increase in rebound burst discharge frequency for Cav3.1 compared to Cav3.2 or Cav3.3 due to differences in window current or activation/recovery time constants. The reduced response by Cav3.2 could optimize its activity where it is expressed in peripheral tissues more subject to temperature variations than Cav3.1 or Cav3.3 channels expressed prominently in the brain. These tests thus reveal that aspects of neuronal firing behavior are critically dependent on both temperature and T-type calcium channel subtype.

**Supplementary Information:**

The online version contains supplementary material available at 10.1186/s13041-021-00813-7.

It is well established T-type calcium channels are important regulators of neuronal firing behavior [1, 2], whereas aberrant T-type channel activity can lead to disorders such as absence seizures and chronic pain [3]. T-type channels are thus considered important pharmacological targets for neurological disorders [3]. The mammalian genome expresses three different T-type channel isoforms (Cav3.1, Cav3.2 and Cav3.3) with distinct distributions in different neuronal populations and unique biophysical and pharmacological characteristics [4–6]. It has been suggested that Cav3 isoforms contribute differentially to the regulation of neuronal excitability [5–8]. Most modeling work is derived from biophysical parameters obtained at room temperature. Our previous work revealed that the gating characteristics of T-type channels are strongly temperature dependent [9]. While Cav3.1 and Cav3.3 channels are expressed predominantly in the brain where temperature fluctuations are small, Cav3.2 channels are also expressed in sensory nerve endings in the skin where they are exposed to much greater temperature variations [3]. It is thus important to determine how different Cav3 isoforms may regulate firing properties of neurons at physiological temperatures. To understand the temperature-dependence of Ca_v_3 kinetics for neuronal output, we built a reduced firing model neuron incorporating the biophysical characteristics of T-type calcium current (*I*_*CaT*_) recorded at 21 °C or 37 °C in our previous study [9] (see Additional file 1).

Additional file 2: Fig. S1 shows that the insertion of *I*_*CaT*_ into the model had different effects on steady-state firing frequency depending on the Ca_v_3 subtype and recording temperature. At 37 °C there was a small decrease in resting firing frequency for each of the Cav3 isoforms compared to 21 °C (Additional file 2: Fig. S1A–C). However, inserting *I*_*CaT*_ into the model greatly increased the ability to generate rebound spike firing following a 70 ms membrane hyperpolarization (Fig. 1). The principal determinant of the duration of burst firing was the inactivation time constant of *I*_*CaT*_. At 37 °C we noted a faster rise to peak in spike frequency during rebound discharge, but also a faster decline back to steady-state frequency for all three channel subtypes (Fig. 1A–C, top traces). An increase in temperature from 21 to 37 °C also increased channel conductance and sped the kinetics of activation and inactivation. The changes in firing frequency closely matched the time course of *I*_*CaT*_, which had faster overall activation and inactivation rates for all three Ca_v_3 isoforms (Fig. 1A–C, lower traces) at 37 °C. Modeling Cav3.1 expression produced the sharpest increase in rebound burst at 21 °C (Fig. 1A, D) compared to only a small rebound burst for Ca_v_3.2 (Fig. 1B, E). This occurred because Ca_v_3.2 had a combination of slow time constants for activation and recovery from inactivation, especially relative to Ca_v_3.1. Ca_v_3.3 also produced a burst response that was faster and of higher frequency  than Ca_v_3.2 because of a larger window current at 21 °C. The major limiting factor to a contribution by Ca_v_3.2 and Ca_v_3.3 to rebound firing at 21 °C was a slow recovery from inactivation, which prevented the current from fully recovering during the preceding 70 ms hyperpolarizing step.

**Fig. 1:**
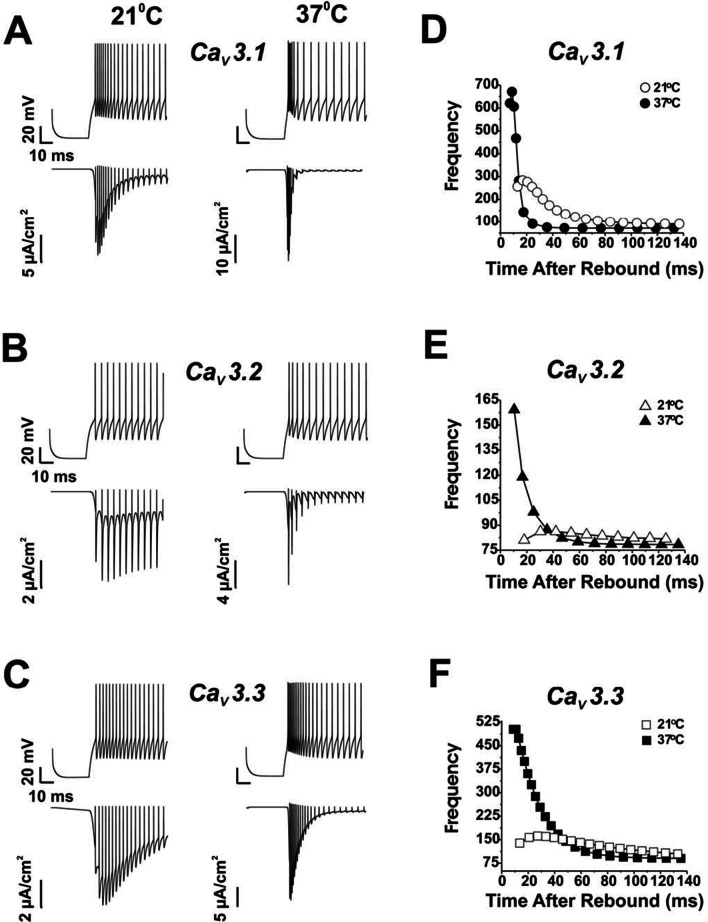
Effects of *I*_*CaT*_ in the model neuron on rebound depolarizations. For each isoform at the two temperatures the model was hyperpolarized (− 5 μA/cm^2^) for 70 ms and then depolarized to 3 μA/cm^2^ to assess the effects of a transient increase in I_CaT_ on firing frequency. **A–C** Neuronal output (top traces) and corresponding inward I_CaT_ current trajectories (lower traces) at the two temperatures for the three Ca_v_3 isoforms. In the right panels (**D–F**) the firing frequencies are plotted as a function of the time since the end of the hyperpolarization for each of the three isoforms at both temperatures. Because these data were derived from mathematical modeling, no error bars are included

Increasing the temperature to 37 °C increased the peak frequency during a rebound burst for all three Cav3 isoforms (Fig. 1A–C). The rate of decrease in firing frequency during the rebound (Fig. 1D–F) was clearly linked to the time course of inactivation of the underlying *I*_*CaT*_ (Fig. 1A–C, lower traces). Furthermore, Ca_v_3.1 and Ca_v_3.3 had much larger peak firing frequencies during rebound bursting using the parameters measured at 37 °C than Ca_v_3.2 because of faster recovery time constants. Thus, Ca_v_3.2 had the least temperature sensitive recovery time constant of the three subtypes, as reflected in its poor ability to change firing frequency following hyperpolarization.

Our results suggest that all three Ca_v_3 isoforms may contribute to rebound depolarizations given the kinetics measured in the HEK cell expression system. Furthermore, the exact spike discharge characteristics elicited during the rebound depolarization may be regulated by the expression of specific Ca_v_3 subtypes [8]. Indeed, a comparison across cell types in cerebellum recorded in vitro revealed that rebound discharge capability was most prominent in cells that expressed the Cav3.1 isoform [8, 10–12]. It has however been difficult to unequivocally attribute a role of T-type channel isoforms to burst activity due to a lack of subtype specific T-type channel inhibitors [13]. Our modeling data provide insights into how individual calcium channel isoforms can regulate neuronal firing behavior according to kinetic parameters and conductance at physiological temperatures.

Our results complement previous data derived from models of thalamic neurons that replaced the biophysical characteristics of native thalamic neuron T-type current(s) with those of T-type isoforms near room temperature to test their contribution to rebound discharge [5, 6]. In Chemin et al. [5], normal thalamic neuron output was most closely mimicked when the native current was substituted with the kinetics of the expressed Ca_v_3.1 current, consistent with the finding that rebound discharge in these cells is abolished by knocking out the Ca_v_3.1 gene [14]. However, substitution of any of the three isoforms enabled some type of spike discharge during the rebound depolarization. In contrast, McRory et al. [6] found that none of the expressed Ca_v_3 kinetics adequately substituted for the native thalamic T-type current, and only Ca_v_3.3 was able to generate a rebound burst discharge. Given the marked dependence of rebound burst discharge on the holding potential and the amplitude and duration of the hyperpolarizing stimulus, it is possible that the model of McRory et al. [5] was not optimized to produce burst firing, particularly with respect to the size of window currents.

As with all models, there are several caveats that cannot be ignored. First, alternate splicing of T-type channels can affect their biophysical characteristics [15], in a manner that could contribute differently to neuronal firing. Second, since most neurons express more than one Ca_v_3 isoform we cannot predict how this might affect the overall neuronal output. Third, we acknowledge that gating characteristics of sodium and potassium channels may also be temperature dependent, but were kept constant in the model. Finally, we reiterate that our model provides only a snapshot of a particular set of experimental conditions, and should be considered a tool that allows one to compare the relative abilities of different T type isoforms to affect firing properties. Nonetheless, our data indicate that T-type channel isoforms differ in their propensities to alter firing rates in neurons in a temperature-dependent manner.

## Supplementary Information

**Additional file 1.** Mathematical modeling. This file describes the mathematical model used to generate Fig. 1.

**Additional file 2: Fig. S1.** Effects of *I*_*CaT*_ on steady-state firing frequency in a model neuron.

## Data Availability

All data generated or analyzed during this study are included in this published article and in our previously published work [9].

## Abbreviation

*I*_*CaT*_: T-type calcium current
