# Supplementary material for: Modeling temperature- and Cav3 subtype-dependent alterations in T-type calcium channel mediated burst firing
Source: Mol Brain. 2021 Jul 17;14:115. doi: 10.1186/s13041-021-00813-7 (PMC8285791; doi:10.1186/s13041-021-00813-7)
Supplement: Supplementary file 1 — Additional file 1. Mathematical modeling. This file describes the mathematical model used to generate Fig. 1. [file 13041_2021_813_MOESM1_ESM.pdf]

Mathematical modeling

All simulations were constructed in MatLab 6.5 using a 4th order Runge-Kutta algorithm with a time step (*dt*) of 0.01 ms. We used a point model described by an electrode current (I_E), a single leak current, a Na^+ current, a K^+ current, and one of three types of transient calcium currents (Ca_v3.1, Ca_v3.2, Ca_v3.3) at either of two temperatures. The kinetic and steady-state properties of each Ca_v3 isoform at both temperatures were individually introduced into the firing model. In addition to I_{CaT}, the model incorporated a standard fast inactivating Na^+ current and a slightly slower K^+ current. The *m*-variable controlling Na^+ inactivation was raised to a power of 3, while the *n*-variable controlling K^+ current activation was raised to the power of 4. The inactivation of Na^+ and activation of K^+ currents were assumed to occur over a similar time scale and modeled using the same dynamical variable, with the *n*-variable being equivalent to 1-*h*. In the absence of I_{CaT} the model had a firing threshold of about -52 mV that occurred with a current injection of 2.15 $\mu A/cm^2$.

For steady-state measurements, the model was driven with a constant current injection of 3 $\mu A/cm^2$. Rebound depolarization was assessed following a brief (70 ms) hyperpolarization of -5 $\mu A/cm^2$ followed by a return to 3 $\mu A/cm^2$. To test the potential effects of temperature-induced changes in Na^+ and K^+ current kinetics, we adjusted the time constants of Na^+ activation, K^+ activation and Na^+ inactivation. In the control model, the activation of Na^+ current was not modeled as a dynamic variable as the Na^+ current activation variable (*m*) was assumed to equilibrate instantaneously with voltage. To test the effects of slowing the Na^+ current activation we thus modeled the *m*-variable as a dynamic variable with time constants ranging from 0.2-0.5 ms. The activation of K^+ current and the inactivation of Na^+ current were modeled by the same dynamical variable, with the *h* and $1 - h$ corresponding to inactivation of Na^+ and activation of

K^+ currents, respectively. The time constant of the *h*-variable was voltage-dependent, but could be changed by multiplying the $\tau(V)$ function by a coefficient ranging from 0.5 – 2. In all cases increasing or decreasing the speed of the Na^+ and K^+ kinetics did not alter the general result of I_{CaT} kinetic changes on rebound burst firing, although the precise spike frequency did change since this is tightly regulated by the kinetics of Na^+ and K^+ currents. This occurred because the time scale of activation of Na^+ and K^+ currents was much faster than that of I_{CaT} inactivation. Thus there exists an effective separation between I_{CaT} and Na^+ and K^+ currents that is related to the differences in time scales of I_{CaT} inactivation and the time constants associated with Na^+ and K^+ currents in the model. Therefore, our model did not incorporate altered Na^+ and K^+ channel gating in relation to temperature changes [1-3]. The activation of each I_{CaT} isoform was then examined in response to the same spike parameters to unequivocally attribute altered neuronal output to the temperature dependent changes in I_{CaT} kinetics. Because the second time constant of inactivation constituted only a small fraction of total inactivation (relative weight of fit parameter compared to the fast inactivation time constant), adding it to the model had a very small effect on burst discharge frequency. In the case of all three subtypes, frequency changes in firing during bursts with a second time constant of inactivation were less than 3% of the maximum firing frequency observed during bursting with a single inactivation time constant. Thus, the model did not incorporate a biexponential inactivation rate. All three Ca_v3 isoforms expressed larger peak currents at 37°C [4], a feature that was not reproduced by only taking kinetics and steady values into account. Currents were thus increased in the model at 37°C by increasing the maximum Ca_v3 conductance (g_{CaT}-variable) to match the experimental results in Fig 1*E*.

The voltage-dependent currents evolved using the following equations:

$$m_\infty = \frac{1}{1+e^{(-(V+40)/3))}}$$

$$\frac{dh}{dt} = \frac{-h+h_\infty}{\tau_h(V)},\ h_\infty = \frac{1}{1+e^{(-(V+40)/-3))}},\ \tau_h(V) = \frac{3248}{\pi(V+95)^2+784}$$

$$\frac{dm_T}{dt} = \frac{-m_T+m_{T\infty}}{\tau_{m_T}(V)},\ m_{T\infty} = \frac{1}{1+e^{(-(V+V_{1/2})/k))}}$$

$$\frac{dh_T}{dt} = \frac{-h_T+h_{T\infty}}{\tau_{h_T}(V)},\ h_{T\infty} = \frac{1}{1+e^{(-(V+V_{1/2})/k))}}$$

The $V_{1/2}$, *k,* and $\tau(V)$ values for $\frac{dm_T}{dt}$ and $\frac{dh_T}{dt}$ functions were taken from our previously reported findings [4]. The time constant-voltage relationships for the T-type currents was not set up using Hodgkin and Huxley formulism, where a single function can be used to describe the time constant-voltage relationship for activation/deactivation and inactivation/recovery time constant. For activation and deactivation we found that no single function could be used to describe both sets of time constants. For this reason, different functions were used to model activation and deactivation. The same approach was used to model inactivation and recovery time constants.

At 21ºC activation and deactivation time constants were formulated as:

Cav3.1

$\tau(V) = 92.3e^{(V/23.6)} + 0.77$ If $V <$ -70 mV, $\tau(V) = 0.139e^{(-V/16.05)} + 0.54$ If $V >$ -70 mV

Cav3.2

$\tau(V) = 53.5e^{(V/21.5)} + 2.37$ If V < -70 mV, $\tau(V) = 0.45e^{(-V/18)} + 0.76$ If $V >$ -70 mV

Cav3.3

$\tau(V) = 1625900e^{(V/7.76)} + 3.54$ If $V <$ -80 mV, $\tau(V) = 0.36e^{(-V/19.9)} + 2.34$ If $V >$ -80 mV

At 37ºC activation and deactivation time constants were formulated as:

Cav3.1

$\tau(V) = 26.7e^{(V/18.26)} + 0.89$ If $V <$ -40 mV, $\tau(V) = 0.0016e^{(-V/4.66)} + 2.95$ If $V >$ -40 mV

Cav3.2

$\tau(V) = 7.05e^{(V/22.8)} + 0.62$ If $V <$ -50 mV, $\tau(V) = 0.004e^{(-V/7.61)} + 0.345$ If $V >$ -50 mV

Cav3.3

$\tau(V) = 85138e^{(V/7.85)} + 1.03$ If $V <$ -70 mV, $\tau(V) = 0.00282e^{(-V/9.61)} + 0.455$ If $V >$ -70 mV

At 21ºC inactivation and recovery time constants were formulated as:

Cav3.1

$\tau(V) = 293e^{(V/86)} + 15$ If $V <$ -80 mV, $\tau(V) = 0.00243e^{(-V/7.27)} + 17.7$ If $V >$ -80 mV

Cav3.2

$\tau(V) = 6687e^{(V/34)} - 90$ If $V <$ -80 mV, $\tau(V) = 0.228e^{(-V/11.41)} + 15.38$ If $V >$ -80 mV

Cav3.3

$\tau(V) = 4748e^{(V/68)} - 651$ If $V <$ -80 mV, $\tau(V) = 0.00266e^{(-V/7.56)} + 66.11$ If $V >$ -80 mV

At 37ºC inactivation and recovery time constants were formulated as:

Cav3.1

$\tau(V) = 232e^{(V/57)}$ If $V <$ -80 mV, $\tau(V) = 0.00319e^{(-V/8.25)} + 2.88$ If $V >$ -80 mV

Cav3.2

$\tau(V) = 2682e^{(V/279)} - 1696$ If $V <$ -80 mV, $\tau(V) = 2.33e^{(-V/34.6)} + 2.63$ If $V >$ -80 mV

Cav3.3

$\tau(V) = 4.7V + 616$ If V < -80 mV, $\tau(V) = 20$ If $V >$ -80 mV

Maximum conductance for the currents, when present, were g_{Na} = 30 μS/cm^2, g_K = 8 μS/cm^2, g_{CaT} = 0.75 μS/cm^2 and g_{leak} = 0.15 μS/cm^2. Reversal values for the currents were E_{Na+} =40 mV, E_{K+} = -90 mV, E_{Ca2+} = 22 mV, and E_{leak} = -65 mV with capacitance (C) = 1 μF/cm^2.

Voltage was integrated according to the equation:

$$C\frac{dV}{dt} = I_E - g_{Na}m_\infty{}^3h(V - E_{Na^+}) - g_K(1-h)^4(V - E_{K+}) - g_{CaT}m_T{}^3h_T(V - E_{Ca^{2+}}) - g_{leak}(V - E_{leak})$$

References:

1. Li H, Liu BG, Dobretsov M, Brull SJ, Zhang JM. Thermosensitivity of large primary sensory neurons. Brain Res. 2002; 926: 18-26.

2. Moran O, Conti F. Properties of the Kv1.1 rat brain potassium channels expressed in mammalian cells: temperature effects. Biochem Biophys Res Commun.1995; 215: 915-920.

3. Ruff RL Effects of temperature on slow and fast inactivation of rat skeletal muscle Na(+) channels. Am J Physiol. 1999. 277: C937-C947.

4. Iftinca M, McKay BE, Snutch TP, McRory JE, Turner RW, Zamponi GW. Temperature dependence of T-type calcium channel gating. Neuroscience. 2006; 142:1031-42.
