# Supplementary material for: Modeling temperature- and Cav3 subtype-dependent alterations in T-type calcium channel mediated burst firing
Source: Mol Brain. 2021 Jul 17;14:115. doi: 10.1186/s13041-021-00813-7 (PMC8285791; doi:10.1186/s13041-021-00813-7)
Supplement: Supplementary file 2 — Additional file 2: Fig. S1. Effects of ICaT on steady-state firing frequency in a model neuron. [file 13041_2021_813_MOESM2_ESM.pdf]

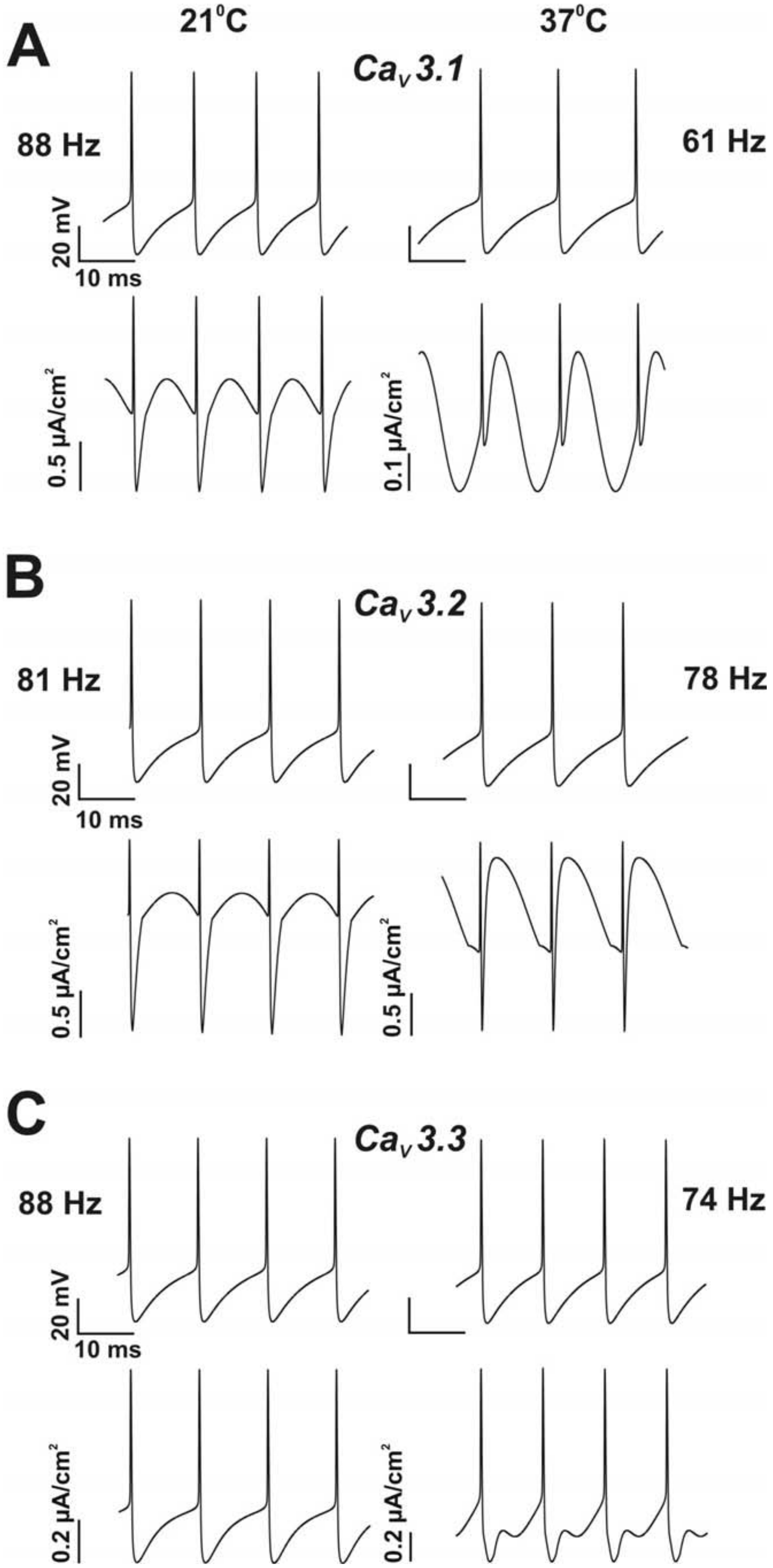
A
21°C
37°C
Cav 3.1
88 Hz
61 Hz
20 mV
10 ms
0.5 µA/cm²
0.1 µA/cm²
B
Cav 3.2
81 Hz
78 Hz
20 mV
10 ms
0.5 µA/cm²
0.5 µA/cm²
C
Cav 3.3
88 Hz
74 Hz
20 mV
10 ms
0.2 µA/cm²
0.2 µA/cm²

FIGURE S1. Effects of I_{CaT} on steady-state firing frequency in a model neuron. The kinetic profiles of the three channel subtypes, $Ca_v3.1$ (***A***), $Ca_v3.2$ (***B***) and $Ca_v3.3$ (***C***), measured at 21ºC and 37ºC were added to the firing model to assess the effects of temperature change on steady-state firing frequency. The model was driven with 3 $\mu A/cm^2$ current injection for 450 ms and frequency was measured by averaging the last 200 ms of spiking to ensure a steady-state measurement. The lower traces in ***A*** ($Ca_v3.1$) , ***B*** ($Ca_v3.2$), and ***C*** ($Ca_v3.3$) show I_{CaT} during steady-state firing at 21ºC and 37ºC.
